# Preterm gut microbiota and metabolome following discharge from intensive care

**DOI:** 10.1038/srep17141

**Published:** 2015-11-24

**Authors:** Christopher J. Stewart, Tom Skeath, Andrew Nelson, Sara J. Fernstad, Emma C. L. Marrs, John D. Perry, Stephen P. Cummings, Janet E. Berrington, Nicholas D. Embleton

**Affiliations:** 1 Faculty of Health and Life Sciences, Northumbria University, Newcastle upon Tyne, United Kingdom; 2 Newcastle Neonatal Service, Royal Victoria Infirmary, Newcastle upon Tyne, United Kingdom; 3 Department of Computer Science and Digital Technologies, Northumbria University, Newcastle upon Tyne, United Kingdom; 4 Department of Microbiology, Freeman Hospital, Newcastle upon Tyne, United Kingdom

## Abstract

The development of the preterm gut microbiome is important for immediate and longer-term health following birth. We aimed to determine if modifications to the preterm gut on the neonatal intensive care unit (NICU) impacted the gut microbiota and metabolome long-term. Stool samples were collected from 29 infants ages 1–3 years post discharge (PD) from a single NICU. Additional NICU samples were included from 14/29 infants. Being diagnosed with disease or receiving increased antibiotics while on the NICU did not significantly impact the microbiome PD. Significant decreases in common NICU organisms including *K. oxytoca* and *E. faecalis* and increases in common adult organisms including *Akkermansia* sp., *Blautia* sp., and *Bacteroides* sp. and significantly different Shannon diversity was shown between NICU and PD samples. The metabolome increased in complexity, but while PD samples had unique bacterial profiles we observed comparable metabolomic profiles. The preterm gut microbiome is able to develop complexity comparable to healthy term infants despite limited environmental exposures, high levels of antibiotic administration, and of the presence of serious disease. Further work is needed to establish the direct effect of weaning as a key event in promoting future gut health.

The preterm gut microbiome has important influences in both the health and disease of neonates^1^. As a result the focus of previous work has been on the role of the bacterial community on the development of necrotising enterocolitis (NEC) and late onset sepsis (LOS)^2^,^3^,^4^,^5^,^6^. While such studies conflict in their findings relating to disease pathogenesis, overall the bacterial diversity in the preterm gut increases over time with a reduction in initial facultative anaerobes including *Staphylococcus* and *Enterococcus* and increases in strict anaerobes including *Bifidobacterium* and *Bacteroides*^7^,^8^. Notably, this increase in strict anaerobes is significantly less than full term, vaginally delivered, breast fed infants over the first 90 days of life^9^.

The gut microbiome of term infants changes during the first year of life, resulting in an ‘adult-like’ profile between 1–4 years^10^,^11^,^12^. *Bifidobacterium* is the genus most differentially represented between childhood and adult profiles^11^. Previous studies suggest that clinical management in early life, such as antibiotic administration, may have profound long terms effects aside from direct gut health, increasing the risk of asthma^13^,^14^, allergy^15^, inflammatory bowel disease^16^,^17^, and obesity^18^,^19^ in childhood and adulthood. However, there is only limited evidence that links such conditions to modulations in early gut microbiome development^20^,^21^,^22^.

A major knowledge gap remains regarding the functional metabolic consequences in the development of the gut microbiome in preterm infants, especially following discharge from the NICU. This may have important consequences for clinical practise within NICUs, long term health, and offer potential interventions that modulate health following discharge. The aim of this study was to explore changes in gut microbiota pre- and post-discharge from the NICU, their relationship to stool metabolomic profiles and any long-term impacts of key neonatal morbidities in preterm infants such as NEC and LOS.

## Results

### Bacterial profiling of the gut microbiota through the NICU and post discharge

PD stool samples from 29 infants aged 1–3 years were analysed as well as the first and last NICU stool samples from 14 of the 29 patients. The PD gut microbiota of infants previously diagnosed with NEC and/or LOS was not significantly different compared to controls (*P* = <0.596). Nor was total days of antibiotic exposure, birth mode (caesarean section or vaginal), gestational age (<or>27 weeks), or age of infant at PD sample collection (stratified as either 12–24 or 25–48 month). Thus, we focus our analysis on the development of the preterm gut microbiome through time.

**Figure 1:**
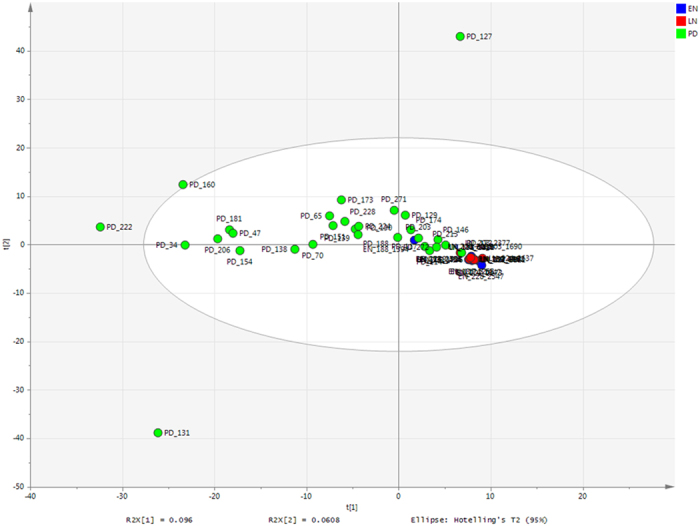
Bacterial profiling PCA of all samples. Coloured according to time point. EN-LN *P* = 0.002; EN-PD *P* = 0.001; LN-PD *P* = 0.001.

**Figure 2:**
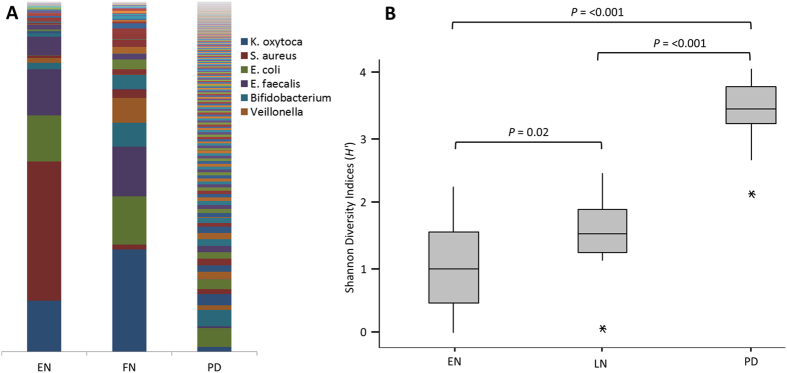
Bacterial community and diversity across the three sample points. (**A**) Bar Chart showing the average bacterial profile at each time point (individual profiles in Supplementary Figure S2). Legend restricted to most abundant OTUs. (**B**) Box plot analysis of the Shannon Diversity index (*H*′) at each time point. EN-Early NICU, LN-Late NICU, PD-Post Discharge.

Maximum parsimony based on the Bray-Curtis showed PD samples were significantly different from EN samples (*P* = 0.001) and LN samples (*P* = 0.001). Within the NICU samples there was also a significant difference (*P* = 0.002) between the EN and LN samples. These significant differences are observed in unconstrained PCA, where the NICU samples are relatively conserved compared to the PD samples, which show a much greater variability in the bacterial profiles (Fig. 1; for only NICU samples see Supplementary Figure S1). The most significant shifts in OTUs from EN to LN included a decreased relative abundance of *S. aureus* (*P* = <0.001) and increased relative abundance of *Veillonella* sp. (*P* = 0.045). Between the LN sample and the PD samples the most significant changes in OTU abundance were decreases in *K. oxytoca* (*P* =  >0.001), *S. aureus* (*P* = >0.001), *E. faecalis* (*P* = >0.001) and increases in *Akkermansia* sp. (*P* = >0.001), Blautia sp. (*P* = >0.001), *Bacteroides* sp. (P = >0.001), and *Collinsella* sp. (*P* = 0.002) (Fig. 2A; for individual profiles see Supplementary Figure S2).

**Figure 3:**
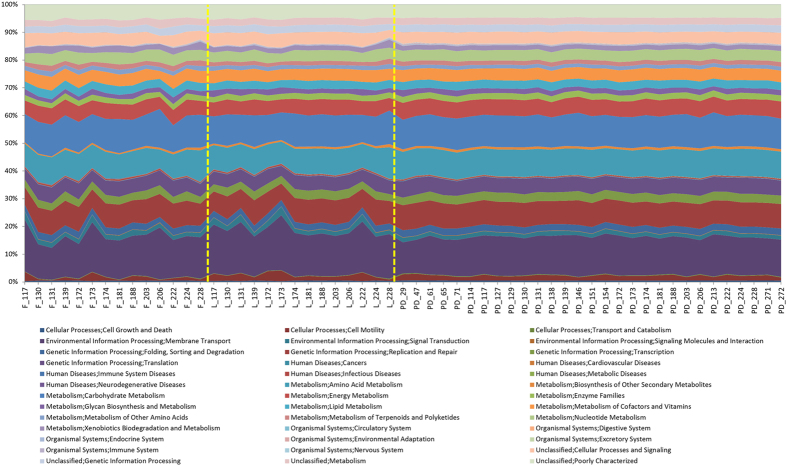
Area chart showing PICRUSt analysis of sequence data. Inferred metagenomic analysis using the data from the next generation sequencing to determine relative abundances of KEGG pathways encoded in the gut microbiota.

A significant increase in the Shannon Diversity index (*H*′) of PD samples compared with EN (*P* = <0.001) and LN (*P* = <0.001) and between the two NICU time points (*P* = 0.02) was also observed (Fig. 2B). Phylogenetic investigation of communities by reconstruction of unobserved states (PICRUSt) was implemented to predict functional content of the bacterial OTUs^23^. This showed that despite greater variation in of the bacterial community, the functional profiles of the PD samples had greater conserved functionality compared to the NICU samples (Fig. 3).

A novel visual inspection analysis of the bacterial profiles supports the above. Visual analysis of EN and LN groups confirmed a decrease of *S. aureus* in LN samples, as well as an increase of *Veillonella sp* (Supplementary Figure S3). The plot also indicates that there may be a decrease of *K. oxytoca* in LN samples although this was not significant in the statistical analysis. Visual analysis of the LN and PD samples showed PD samples are generally more diverse with higher levels in more OTUs (Supplementary Figure S4). However, A decrease in *K. oxytoca*, *S. aureus* and *E. faecalis* occurred from LN to PD, as well as *Clostridium sensu stricto*, although this was not significant in the main statistical analysis. As confirmed by the statistical analysis *Akkermansia sp*, *Blautia sp*, *Bacteoides sp* and *Collinsella sp* (are all increased in PD samples. However, these represent overall trends and not all samples followed this pattern.

### Metabolomic profiling of the preterm gut through the NICU and post discharge

**Figure 4:**
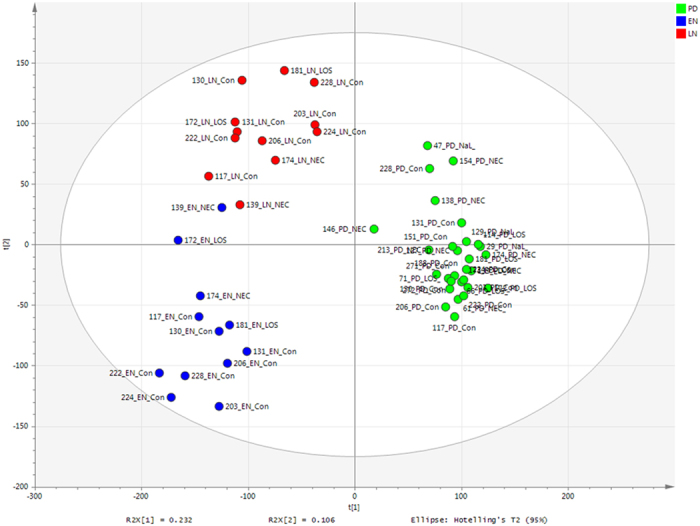
PLS-DA score scatter plot of Metabolomics raw data (R2Y = 89%). PLS-DA loadings-EN R2 0.85, Q2 0.73; LN R2 0.84, Q2 0.67; PD R2 0.96 and Q2 0.93.

**Figure 5:**
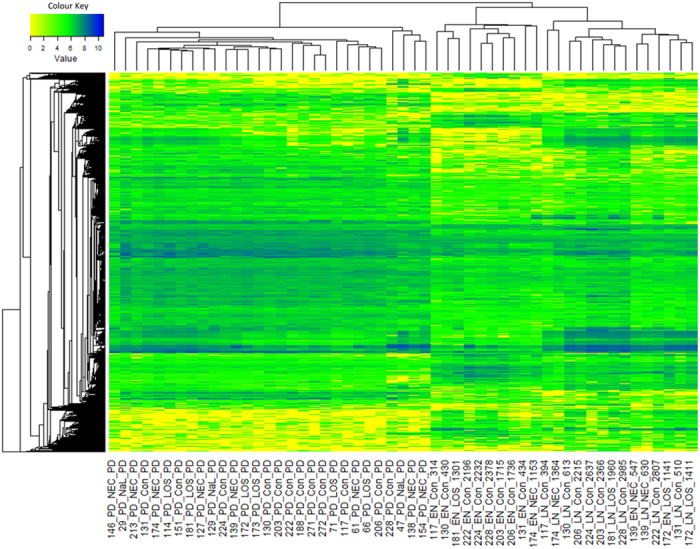
Metabolomics heatmap of all samples. All values were log10 transformed.

PLS-DA of metabolomic profiling data showed the grouping by time point to be highly predictive (EN R2VY = 0.85 and Q2VY = 0.73, LN R2VY = 0.84 and Q2VY = 0.67, and PD R2VY = 0.96 and Q2VY = 0.93, where <0.5 represents well modelled reproducible variables) (Fig. 4). Following removal of noise (metabolites clustered around the origin and not associated with a given group), the metabolite profiles cluster almost exclusively based on time point, with the exception of EN samples from patients 139 and 172 which group with the LN samples (Fig. 5). However, analysis of *only* PD samples supervised according to NICU demographics including birth mode, gestational age, age at sample collection, days of antibiotics, or diagnosis of NEC and/or LOS did not show robustly modelled variables and were thus deemed not significant (e.g. Supplementary Figure S5), in accordance with the next generation sequencing data.

With the threshold used in SIEVE, 51,058 components (metabolites) were detected in total with the majority (47,631) being detected in all samples, regardless of time point (Supplementary Figure S6a). However, a general trend showed the lowest overall intensity in the EN samples and the highest intensity in PD samples. Although relatively low numbers of metabolites were shown to be specific to each group, there were 1,492 components only present in the LN and PD groups and a further 473 components only present in the PD group (Supplementary Figure S6b). No accurate and robust identification of significant metabolites associated with each time point could be determined.

## Discussion

The aim of this study was to explore a crucial knowledge gap in the longitudinal development of the preterm gut microbiome following discharge from the NICU. We utilised two complementary technologies in bacterial 16S sequencing and metabolomics to more completely characterise microbiome changes. Because of the long timeframe needed to accrue sufficient numbers of very preterm infants surviving NEC or LOS, the PD samples in this study came from infants with ages ranging from 1–3 years, but all were consuming normal solid diets. We explored potential differences between the PD samples stratified by age and detected no significant difference and thus analysed them collectively.

A significant reduction in the relative abundance of *S. aureus* (*P* = <0.001) and an increased relative abundance of *Veillonella* sp. (*P* = 0.045) was found between the EN and LN periods. In accordance with previous studies, the highest levels of *S. aureus* in EN was found in patients delivered by caesarean section, with bacterial signatures of delivery mode lost in the LN samples prior to discharge^24^. While the bacterial diversity remained relatively low during NICU stay, there was a significant increase in diversity from EN to LN (*P* = 0.02), indicating the establishment of more complex gut communities in the NICU, despite restricted exposure to microorganisms^8^. It has previously been suggested that preterm infants undergo delayed establishment of the gut microbiome with lower overall diversity over the first 3 months of life^25^. However, the bacterial diversity was found to significantly increase following discharge (*P* = <0.001), with diversity scores comparable to those of full term infants^20^.

PCA analysis of the bacterial profiles showed that the NICU samples cluster together independent of time point, however the PD samples show a high degree of variation. It is noteworthy that significant changes between the relative abundance of species occurring during the NICU period are comparably minor compared to the much great complexity and individual nature of an infant gut microbiota following discharge. The most significant *decreases* in relative abundance PD corresponded to *K. oxytoca* (*P* = >0.001), *S. aureus* (*P* = >0.001), and *E. faecalis* (*P* = >0.001), which are organisms typically associated with the NICU environment^6^,^26^. The significant reduction in these organisms probably represents increased exposure to non-hospital environmental microbes and a more complex diet^10^. The most significant *increases* in relative abundance in PD samples corresponded to *Akkermansia* sp. (*P* = >0.001), *Blautia* sp. (*P* = >0.001), *Bacteroides* sp. (P = >0.001), and *Collinsella* sp. (*P* = 0.002). Importantly, these genus are all common constituents of the healthy adult gut microbiota and their presence further suggests that the potentially vulnerable preterm gut is able to achieve a flora comparable to that of a healthy full term infant^11^. The reduction of previously abundant NICU organisms and increases in organism more common to the healthy adults gut microbiota is interesting and might represent the influence of more complex diet. For example, members of the genus *Bacteroides* are capable of utilising plant derived polysaccharides and by the PD stage of life in this study infants will be consuming complex solid diets^27^. Furthermore, *Blautia* was recently only detected following weaning in a piglet model^28^.

In contrast to the bacterial data, the metabolomic profiles showed a relatively high degree of comparability between the PD samples. The majority of metabolites were detected at each time point, suggesting functional aspects of preterm gut microbiome development is uniform, as oppose to undergoing significant stepwise changes after discharge. An increase in the number and intensity of identified metabolites correlated with increased life stages^25^. This is similar to data using gas chromatography MS (GCMS), where the number of detected volatile organic compounds (VOCs) were shown to increase with increasing age in a comparable cohort of infants^29^.

The gradual progression of the gut metabolome corresponds with the microbiota data presented here and in previous studies, where the bacterial richness increased overtime, rather than new species replacing existing species^4^. The striking observation of PD samples to have conserved metabolic function profiles, both for specific inferred bacterial function (PICRUSt analysis) and overall gut function (LCMS analysis), despite distinct and variable gut microbiota profiles, is important. This is in accordance with previous studies using metagenomic analysis which show un-weaned infants (i.e. not consuming complex semi-solid foods) had a relatively simple gut microbiota, but high variation in functional composition in comparison to weaned infants and adults who had more a complex gut microbiota but greater functional uniformity, regardless of age or sex^30^,^31^. Such data suggests that the human gut requires a relatively conserved suite of functions and the actual species colonizing the host and their relative abundance can vary, providing the individual has the overall functional capacity demonstrated here.

No significant long-term modifications to the gut microbiota were found in response to all patient variables tested. The age of the infant at PD sampling did not have a significant effect on the gut microbiome, further suggesting the potential importance of sentinel events much earlier in infancy, such as weaning. We further show that diagnosis with serious disease (NEC and/or LOS) and the associated increased in antibiotic exposure did not have significant long-term effects on the gut microbiome. Further studies with larger numbers of infants are needed to describe how early alteration of the gut microbiome effects long-term inferences on the host, such as obesity^19^.

Although this study shows the preterm gut microbiome develops in a similar fashion to healthy full term infants, it is still unclear at which age an infant gut microbiome stabilises and remains comparable throughout adulthood. One emerging hypothesis is that the introduction of semi-solid foods is a key event where the gut microbiome develops into its adult form. Thus further work should focus sampling around and subsequently to these events as this crucial period may represent a ‘window of opportunity’ at which modification of the gut microbiota (eg with probiotics/prebiotics etc.) can influence long-term health^32^,^33^. Future follow-up work using carefully collected cohorts of preterm and term infants with detailed pheno- and entero-typing and demographic data throughout life presents the exciting possibility to explore how the early gut microbiome development influences growth, development, and health and disease throughout maturity.

In summary, we show that the preterm gut microbiome undergoes significant changes following discharge from the NICU, with an increase in diversity and accumulation of organisms comparable to that of term infants, regardless of earlier disease and limited environmental exposure. Furthermore, while increased complexity and individualisation of the gut microbiota correlated with increased age, the overall functional properties of the gut metabolome become more stable and conserved between infants.

## Methods

### Ethics statement

Ethics approval was obtained from the County Durham and Tees Valley Research Ethics Committee and written informed parental consent was obtained. The methods were carried out in accordance with the approved guidelines.

### Infants and samples

Infants who had enrolled to our SERVIS study were approached in a selective manner and asked to provide a stool sample in later infancy. Infants were selected on the basis of previous disease state (NEC and/or LOS) and gestationally matched controls. Fifty-five infants were identified based on all previous NEC (*n* = 20) and LOS (*n* = 15) cases and sufficient controls (*n* = 20). Of the 56, 29 contributed a post discharge stool (8 NEC, 6 LOS, 3, NEC and LOS, and 12 controls) between 1 and 3 years of age (Table 1). Post discharge (PD) stool samples were sent using standard postal systems and stored at −20 °C immediately upon receipt and then at −80 °C.

Additional analysis was carried out on an early NICU (EN) and late NICU (LN) stool pre discharge from 14 infants (*n* = 28), where detailed sequencing data was available from patients involved in another study (MG-RAST accession numbers 4603816.3–4603844.3). 16S rRNA bacterial profiling was performed on all samples in the study. Metabolomics was carried out on all samples, with the exception of the NICU samples from patients 173 and 188, where insufficient informative sample remained. The unit has standardised feeding practices, antibiotic and antifungal use and no infant was supplemented with probiotics during their NICU stay.

### 16S rRNA Bacterial profiling

Nucleic acid extraction of stool was carried out on 100 mg of sample using the PowerLyzer™ PowerSoil® DNA Isolation Kit (MoBio, CA, USA) in accordance with the manufacturer’s instructions. Bacterial profiling utilised the 16S rRNA gene targeting variable region 4 (V4) and was carried out by NU-OMICS (Northumbria University) based on the Schloss wet-lab MiSeq SOP and raw fastq data were processed using Mothur (version 1.31.2) as previously described^34^. Chimeric sequences were detected by Chimera.uchime and removed from downstream analysis. Alignment was generated via the Silva database^35^. A cutoff of 70 was applied to assign sequences to the trainset9_032012 resulting in 5,496,973 reads. All sequences from PD samples were deposited in MG-RAST under the accession numbers 4516545.3–4516585.3.

### LCMS/MS metabolomic profiling

Water, methanol, and acetonitrile (ACN) were liquid chromatography mass spectrometry (LCMS) grade (VWR). Metabolites were extracted from 100 mg stool, homogenised in 1 mL cold 80% methanol by vortexing for 15 min at 4 °C. The suspension was then centrifuged at 10,000 × g for 10 min at 4 °C and lyophilised in a freeze dryer before storage at −80 °C. Samples were re-suspended in 1 mL of initial start phase buffer (5% ACN). Stool metabolite profiling was performed using reverse-phase ultra-performance LCMS tandem mass-spectrometry (UPLC-MS/MS). An Accucore C18 column (2.6 μm, 150 × 2.1 mm) was used at 40 °C with a 3.0 μl injection and 300 μl/min flow rate throughout. A multi-step LC gradient was used with 5% ACN increasing to 95% ACN over 22 minutes, with a further 95% ACN for 3 minutes followed by a final 5 minutes re-equilibration at 5% ACN. Samples were run in triplicate and the order of samples in each triplicate sequence was randomised. A blank consisting of LCMS grade water underwent the same procedure and an aliquot of every sample was used as a pool. Prior to each run a blank and 5 pools were processed to equilibrate the system and then blanks and pools were processed periodically every 10 samples. A Q-Exactive (Thermo) was used for the MS and subsequent data dependant MS/MS. Metabolomic profiling was performed using HESI with high resolution (70,000) positive and negative switching. The mass range was set from 100–1000 m/z. MS/MS was subsequently employed with data dependency based on the metabolites of interest.

SIEVE (Version 2.2 *beta*) was used to process the Thermo RAW files by component extraction. All blanks were used for background subtraction. Positive and negative data were processed individually and combined prior to downstream analysis. An intensity threshold of 500,000 was applied detecting a total of 51,058 components (36,405 positive and 14,653 negative). Data dependant MS/MS (ddMS2) analysis of significant components was performed for putative identification and monoisotopic masses were matched against inhouse standards, however accurate metabolite identification could not be obtained.

### Statistical analysis

For normalisation of the NGS bacterial profiling data, all samples were subsampled to 35,448 reads and singleton reads were removed. Significance of variables in the NGS data was analysed within Mothur using maximum parsimony based on the Bray-Curtis, where *P*-values of <0.05 were deemed significant. Bacterial profiling data was analysed by principal component analysis (PCA) using SIMCA 13.0 (Umetrics, Stockholm, Sweden)^36^. Metabolomic profiling data was analysed using partial least squared-discriminatory analysis (PLS-DA). Operational taxonomic units (OTUs) and components were automatically log transformed within SIMCA. Only variables with a VIP of >1 were included and the most significant variables associated with each group were determined based on the variables with error bars above 0 in the coefficient plot^37^. Multiple iterations of the VIP analysis were conducted for optimal noise reduction (Supplementary Figure S7). To check that data was adhering to multivariate normalities, Hotelling’s *T*^*2*^ tolerance limits were calculated and set at 0.95. Heatmaps were generated in R using the vegan and gplots packages.

The bacterial profiles of the samples were further visually investigated using a Parallel Coordinates plot^38^, where each axis represents an OTUs and the bacterial profile of a sample is represented by a polyline intersecting the axes at corresponding bacterial level. The sample profiles are coloured according to sample classification to support comparison between sample groups. The profiles are log transformed and display the 40 most abundant OTUs.

## Additional Information

**How to cite this article**: Stewart, C. J. *et al.* Preterm gut microbiota and metabolome following discharge from intensive care. *Sci. Rep.* **5**, 17141; doi: 10.1038/srep17141 (2015).

## Supplementary Material

Supplementary Table S1

Supplementary Table S2

Supplementary Information

## Figures and Tables

**Table 1:** Patient demographics.

Patient	Diagnosis^1^	Birth Weight	Gestation Age	Birth Mode	Breast Milk	First Breast Milk (DOL)	Full Feed 72 hrs (DOL)	NICU Antibiotics (days)	Age at sampling		
									Day of EN^2^	Day of LN^2^	Month of PD
29	N S	800	25	V	Y	3	34	16	–	–	48
47	N S	760	26	V	Y	3	15	43	–	–	46
61	N	1030	27	V	Y	2	14	20	–	–	42
66	S	630	24	V	Y	3	22	32	–	–	41
71	S	860	26	V	Y	11	21	12	–	–	33
114	S	750	25	V	Y	1	7	21	–	–	32
117	C	870	26	CS	Y	2	10	2	12	60	26
127	N	1600	31	V	Y	1	19	24	–	–	25
129	N S	700	24	V	Y	3	35	31	–	–	28
130	C	1000	27	CS	Y	4	15	15	8	83	28
131	C	545	27	CS	Y	4	27	7	10	54	23
138	N	804	26	CS	Y	7	28	19	–	–	27
139	N	1470	30	CS	Y	2	10	6	8	42	26
146	N	1195	28	V	Y	2	14	13	–	–	23
151	C	1020	27	V	Y	1	Missing	32	–	–	25
154	N	1060	28	V	Y	1	7	19	–	–	25
172	S	1060	28	V	Y	1	10	9	8	50	23
173	S	1150	28	CS	Y	2	18	16	2	34	23
174	N	1350	29	CS	N	N/A	15	16	11	48	23
181	S	570	23	V	Y	4	14	2	8	88	22
188	C	750	24	V	Y	2	39	13	4	94	22
203	C	1130	26	V	Y	1	15	5	2	68	20
206	C	1255	28	V	Y	0	11	7	4	51	20
213	N	1120	28	CS	N	N/A	9	6	–	–	20
222	C	620	24	V	Y	3	21	15	5	86	19
224	C	1170	28	V	Y	2	24	4	4	51	19
228	C	910	25	V	Y	4	14	34	3	94	19
271	C	2030	31	V	Y	2	8	2	–	–	14
272	C	1535	31	V	Y	2	7	2	–	–	14

^1^N – Necrotising enterocolitis, S – late onset sepsis, C – control.

^2^represents no NICU sample was obtained from the patient.
