# Supplementary material for: Preterm gut microbiota and metabolome following discharge from intensive care
Source: Sci Rep. 2015 Nov 24;5:17141. doi: 10.1038/srep17141 (PMC4657104; doi:10.1038/srep17141)
Supplement: Supplementary Information [file srep17141-s3.pdf]

Supplementary Information

Preterm gut microbiota and metabolome following discharge from intensive care

Christopher J Stewart[1,*], Tom Skeath[2], Andrew Nelson[1], Sara J Fernstad[3], Emma CL Marrs[4], John D Perry[4], Stephen P Cummings[1], Janet E Berrington[2], Nicholas D Embleton[2]

[1]Faculty of Health and Life Sciences, Northumbria University, Newcastle upon Tyne, United Kingdom

[2]Newcastle Neonatal Service, Royal Victoria Infirmary, Newcastle upon Tyne, United Kingdom

[3]Department of Computer Science and Digital Technologies, Northumbria University, Newcastle upon Tyne, United Kingdom

[4]Department of Microbiology, Freeman Hospital, Newcastle upon Tyne, United Kingdom

*christopher.stewart@northumbria.ac.uk

Supplementary Table S1 – Raw 16S profiling dataset

Supplementary Table S2 – Raw LCMS metabolomics dataset

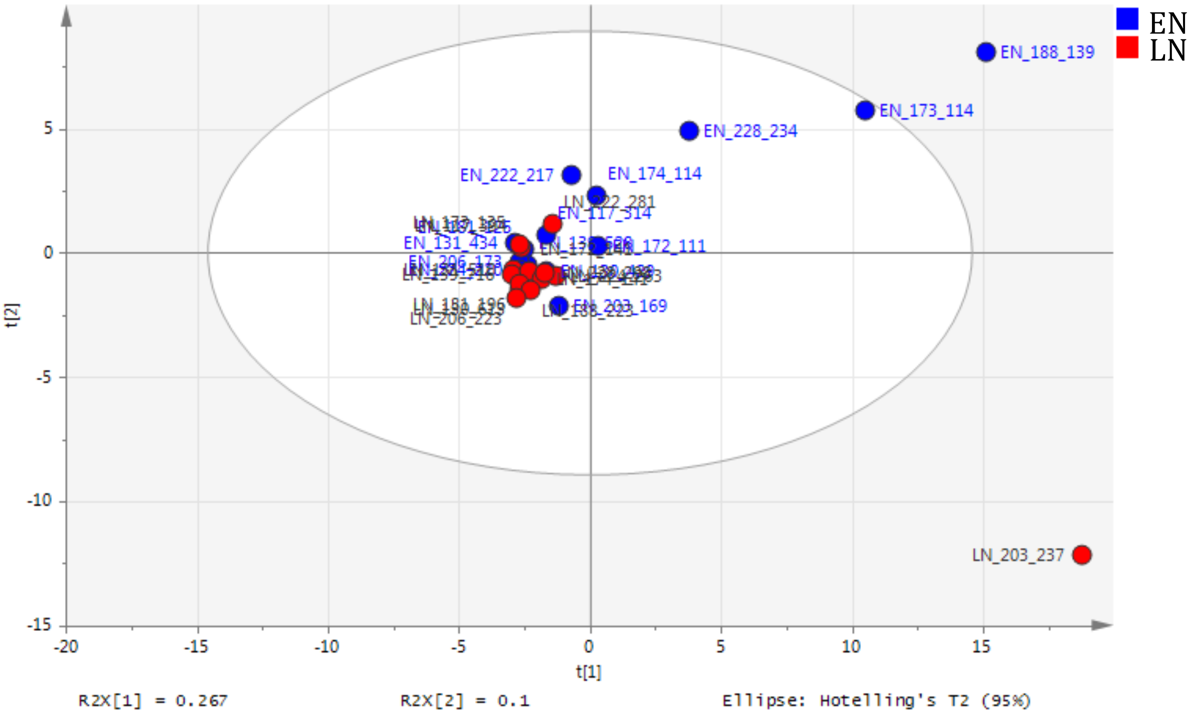

Supplementary Figure S1 - Bacterial profiling PCA of only NICU samples. Coloured according to time point. EN-LN $P = 0.002$.

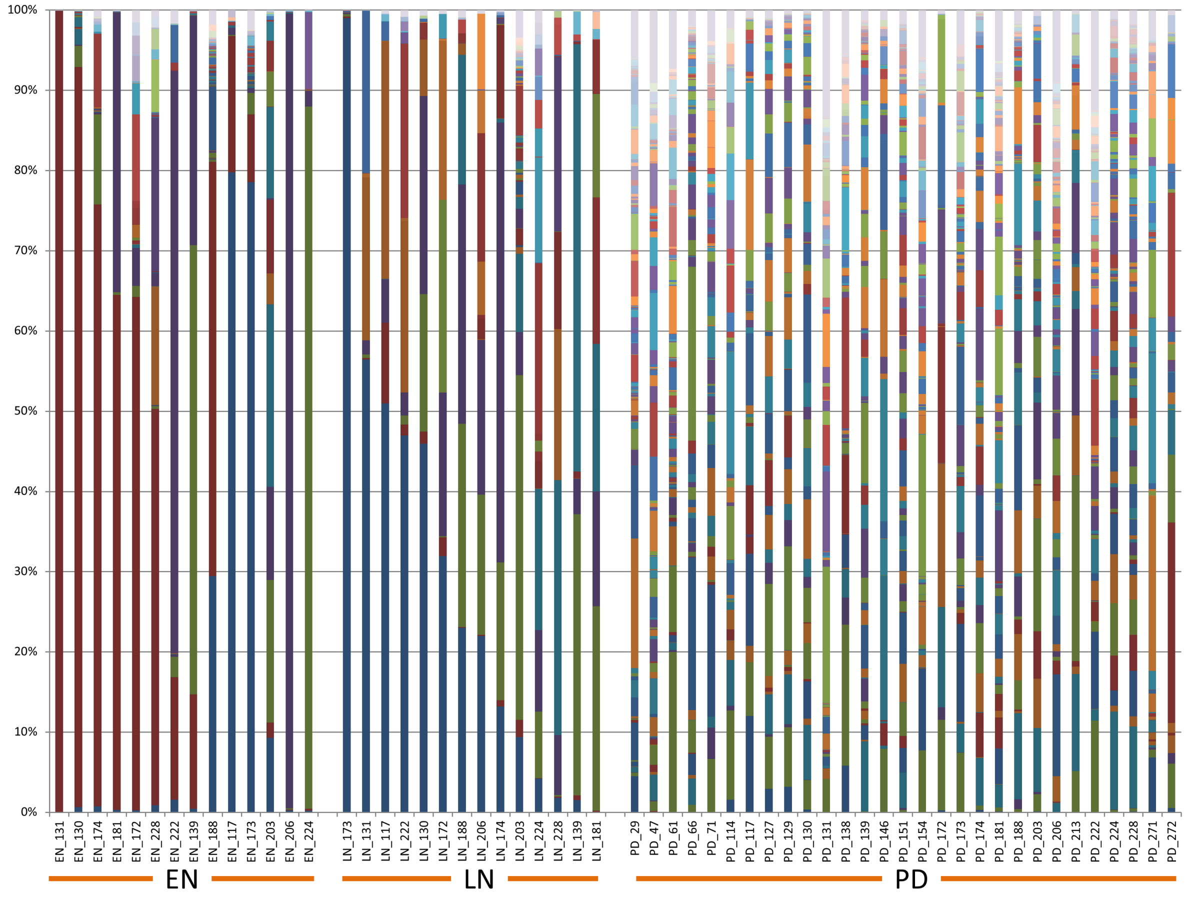

Supplementary Figure S2 – Bacterial profiling bar charts of individual samples at each time point.

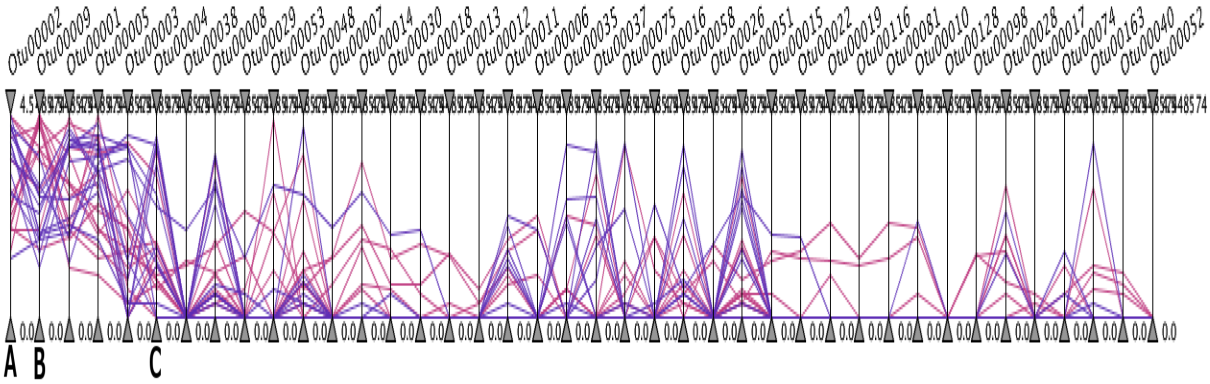

Supplementary Figure S3 – Parallel Coordinates displaying the bacterial profiles of EN (pink) and LN (purple) samples for the 40 most abundant OTUs. Letters indicate OTUs displaying increase or decrease from EN to LN. A = *S. aureus*, B= *Veillonella* sp., C = *K. oxytocea*.

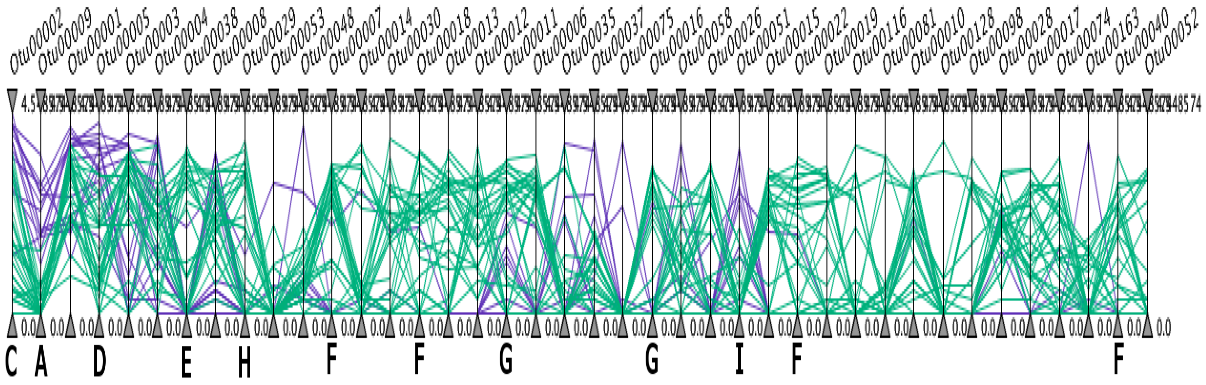

Supplementary Figure S4 - Parallel Coordinates displaying the bacterial profiles of LN (purple) and PD (green) samples for the 40 most abundant OTUs. Letters indicates OTUs displaying increase or decrease from LN to PD. A = *S. aureus*, C = *K. oxytocea*, D = *E. faecalis,* I = *Clostridium sensu stricto*, E = *Akkermansia sp*, F = *Blautia sp*, G = *Bacteoides sp*, H = *Collinsella sp*.

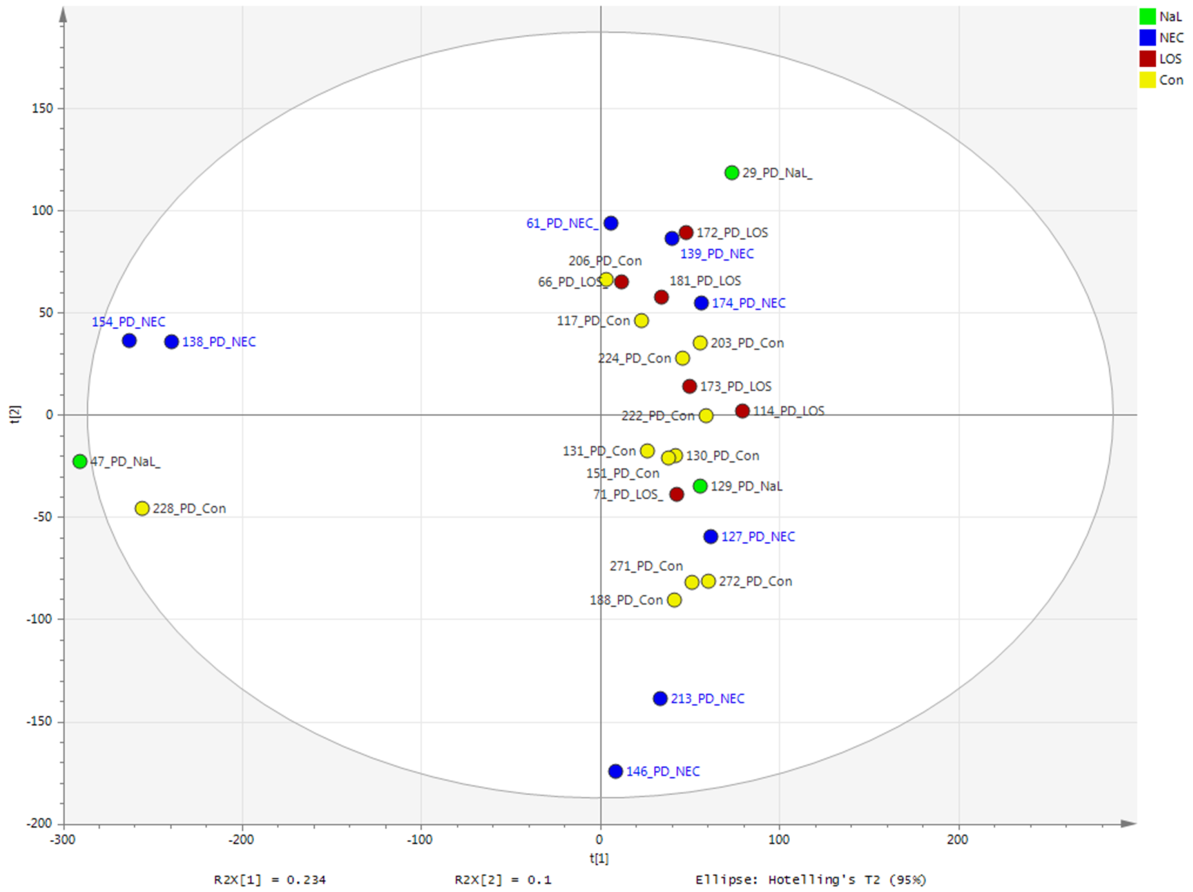

Supplementary Figure S5 – Metabolomic profiling PCA score plot and PLS-DA loadings of post discharge samples stratified according to previous disease diagnosis. A) PCA. PLS-DA Loadings – NaL R2 0.46, Q2 -0.09; NEC R2 0.12, Q2 -0.12; LOS R2 0.14, Q2 -0.07; CON R2 0.60, Q2 -0.00.

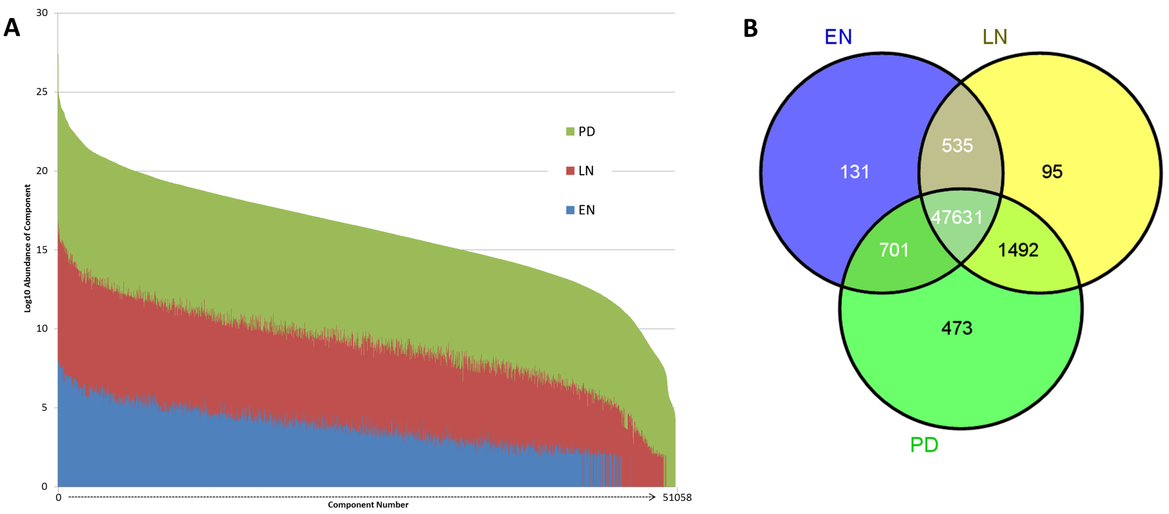

Supplementary Figure S6 – Number and relative intensity of metabolites between the groups. A) Stacked bar chart comparison showing the average log10 abundance of each metabolite detected at each time point. B) Venn diagram showing the presence and absence of metabolites across the groups.

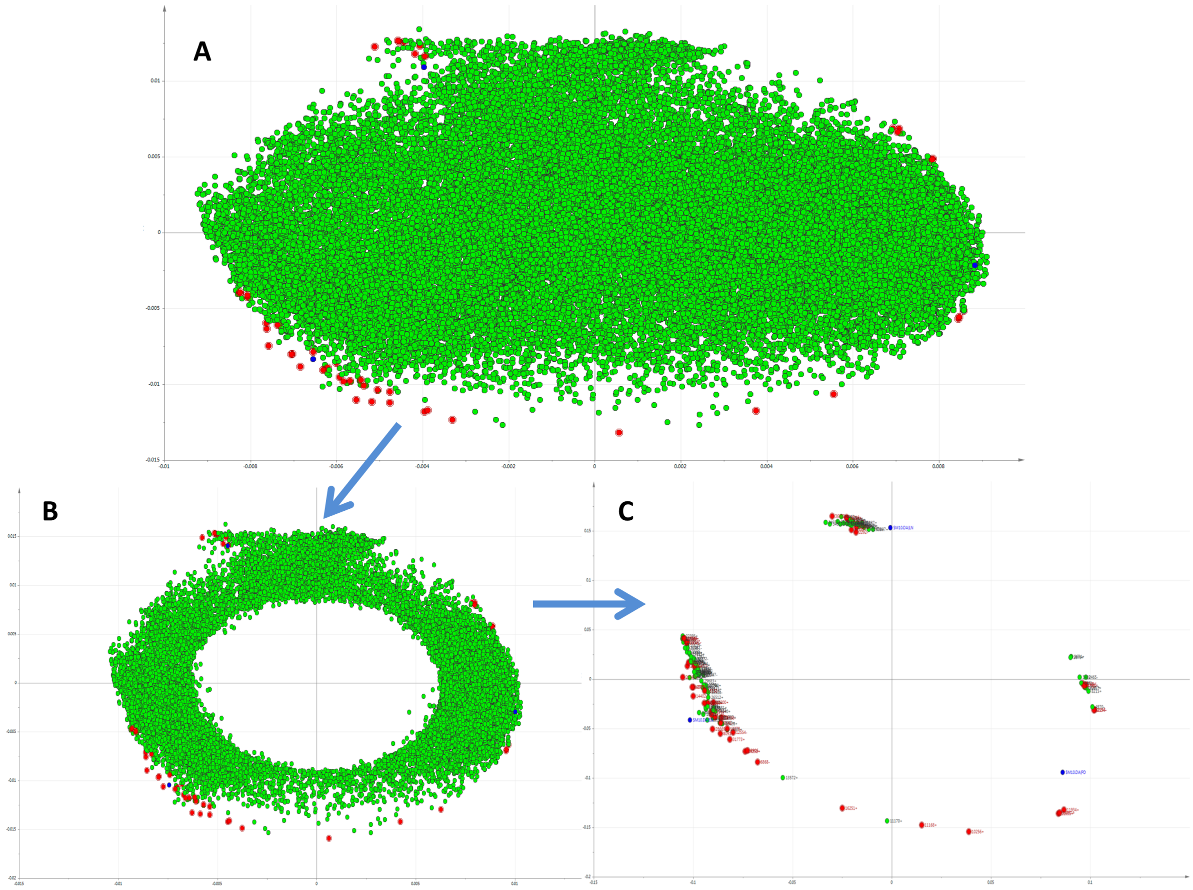

Supplementary Figure S7 – PLS-DA Loadings through multiple iterations of 'VIP' to remove noise. Blue circles represent the groups; EN – Early NICU, LN – Late NICU, PD – Post Discharge, Green circles are component where red components show the most significant metabolites which underwent MS2. A) Raw data. B) First round of VIP. C) Final loadings.
